# Effects of different crop intercropping on quality and rhizosphere soil microbial community of *Erigeron breviscapus*

**DOI:** 10.3389/fmicb.2026.1834232

**Published:** 2026-08-17

**Authors:** Tiantao Wang, Shicha Chen, Zuyun Zhang, Dejun Guan, Jianing Feng, Ping He, Jia Mo, Ruopeng Yang

**Affiliations:** 1 College of Agricultural Sciences, Honghe University, Mengzi, China; 2 Yunnan Provincial Key Laboratory of Cross-Border Chinese Herbal Materials, Mengzi, China; 3 Yunnan Zesheng Biological Technology Co., Ltd., Luxi, Yunnan, China; 4 College of Biological and Food Engineering, Honghe University, Mengzi, China

**Keywords:** enzyme activity, *Erigeron breviscapus*, intercropping, morphology, rhizosphere microorganisms, root system

## Abstract

Obstacles for continuous cropping severely restrict the sustainable production of *Erigeron breviscapus*. To screen suitable intercropping crops and clarify their dual effects on plant growth, medicinal quality, rhizosphere nutrients, soil enzyme activity, and microbial communities, a pot experiment was conducted using 3-year continuously cropped soil, with monoculture as the control (CK) and four intercropping treatments: (A) *Brassica juncea* L., (B) corn, (C) purple garlic, and (D) broad bean. The results showed distinct crop-specific positive and negative effects of intercropping. On the positive side, intercropping with corn and broad bean significantly increased the total flavonoid content; corn and purple garlic intercropping elevated soil catalase and phosphatase activities, while all four intercropping modes improved soil pH, organic matter, and available nutrients and significantly enriched the richness and diversity of rhizosphere bacterial communities, except in the purple garlic treatment. A correlation analysis indicated that the beneficial phylum *Actinobacteriota* was positively correlated with soil pH and hydrolase activities, and intercropping effectively reduced the relative abundance of pathogenic *Fusarium*. However, substantial negative drawbacks were observed across all intercropping systems. All intercropping treatments suppressed aboveground leaf development and root morphological indicators of *E. breviscapus*, and the scutellarin content was significantly lower than CK at all sampling stages. Intercropping with purple garlic drastically reduced fungal community richness and the number of unique fungal operational taxonomic units, which may weaken soil disease-suppressive capacity. Although corn and broad bean partially alleviated rhizosphere microecological degradation caused by continuous cropping, they simultaneously induced irreversible declines in vegetative growth and key medicinal component accumulation. In conclusion, no fully balanced optimal intercropping combination was identified in this pot experiment; corn and broad bean showed partial potential for soil restoration under controlled pot culture conditions; however, their adverse impacts on medicinal quality and plant growth cannot be ignored. Large-scale field positioning trials are urgently required to verify these pot-derived findings and optimize intercropping configuration, planting density, and cultivation regulation before the coordinated intercropping scheme can be applied to field *E. breviscapus* production to balance soil health, crop growth, and medicinal efficacy.

## 1 Introduction

*Erigeron breviscapus*, also known as *Erigeron breviscapus var. brevicaulis*, is a perennial herb belonging to the genus *Erigeron* in the family Asteraceae (formerly Compositae) (Du et al., 2025). Its roots, stems, and leaves all possess high medicinal value. Extracts of *Breviscapine* have been reported to alleviate problems associated with some cardiovascular diseases (Lin et al., 2024), diabetes (Zhang et al., 2020), pulmonary arterial hypertension, and platelet regulatory disorders (Dong and Qu, 2019; Wen et al., 2021), while also enhancing liver antioxidant capacity. However, the *E. breviscapus* industry in Yunnan Province has faced various problems, including the lack of appropriate seeds and continuous cropping obstacles. Continuous cropping obstacles can reduce soil microbial diversity, promote the accumulation of autotoxic substances, and alter soil physicochemical properties, thereby affecting crop yield and quality (Muhammad et al., 2025; Huang et al., 2025; Shen et al., 2024). Thus, continuous cropping has become the most significant problem facing the *E. breviscapus* industry.

Intercropping is a planting system that uses root exudates from intercropping plants to alter the rhizosphere microecological environment of continuously cropped plants, which enables the modification of the microbial community to mitigate continuous cropping obstacles, thereby promoting crop growth (Gao and Zhang, 2023; Grace et al., 2025). By simultaneously planting different types of plants, intercropping not only helps with pest control but also optimizes nutrient supply and maximizes land space utilization (Anjaharinony et al., 2023; Li et al., 2021; Wang et al., 2025). Moreover, intercropping can increase the effective nutrient content of soil, enhance soil enzyme activity, enrich microbial species diversity, and reduce the occurrence of soil-borne diseases. Numerous studies have shown that the use of allelopathy between plants can not only increase crop yield and quality but also effectively reduce the development of pests and diseases, improve the soil ecological environment, and reduce the occurrence of continuous cropping obstacles (Akhtar et al., 2024). Liu et al. (2024) used high-throughput sequencing and metabolomics to assess the microbial community structure and the composition and abundance of metabolites in the rhizosphere soil of *Perilla frutescens* L. Britt. (perilla) after different continuous cropping years. The results indicated that differential metabolites in soils with different durations of continuous cropping were primarily associated with pathways involved in cellular lipid formation, oxidative stress responses, and energy metabolism. Tang et al. (2022) indicated that allelochemicals induce dysfunction of the root systems of peanut crops, leading to impaired osmotic balance, decreased root enzyme activity, stunted crop growth, and reduced yield. However, a majority of the studies only analyze the effects of continuous cropping obstacles on crops and on identifying crops most suitable for intercropping. Very few studies have analyzed the effects of compatible intercropping crops on the morphological growth and medicinal active components of the target crops. Crop intercropping can alter the structure of microbial communities and reduce the occurrence of soil-borne diseases (Olatunde et al., 2026). Therefore, investigating the impact of intercropping on soil health from a microbial perspective is important. Meanwhile, it is also imperative to clarify whether the rhizosphere microbial community of intercropping-planted plants differ from those of monocultured plants. Therefore, to address the continuous cropping obstacles of *E. breviscapus*, this study aimed to analyze the effects of intercropping different crops with *E. breviscapus* on soil quality, the structure of the rhizosphere microbial community of *E. breviscapus*, its morphological growth, and the quality of medicinal active components. Ultimately, the study aimed to determine the optimal compatible crops for addressing the continuous cropping obstacles of *E. breviscapus* in Yunnan Province.

## 2 Materials and methods

### 2.1 Experiment materials

*Erigeron breviscapus* (Zesheng Co., China) was the main crop used in this study, and the intercropping crops included corn (Qinrui 189), purple-skinned garlic, broad bean (Yundouzao 16), and *Brassica juncea* L.

### 2.2 Experiment design

**Figure 1:**
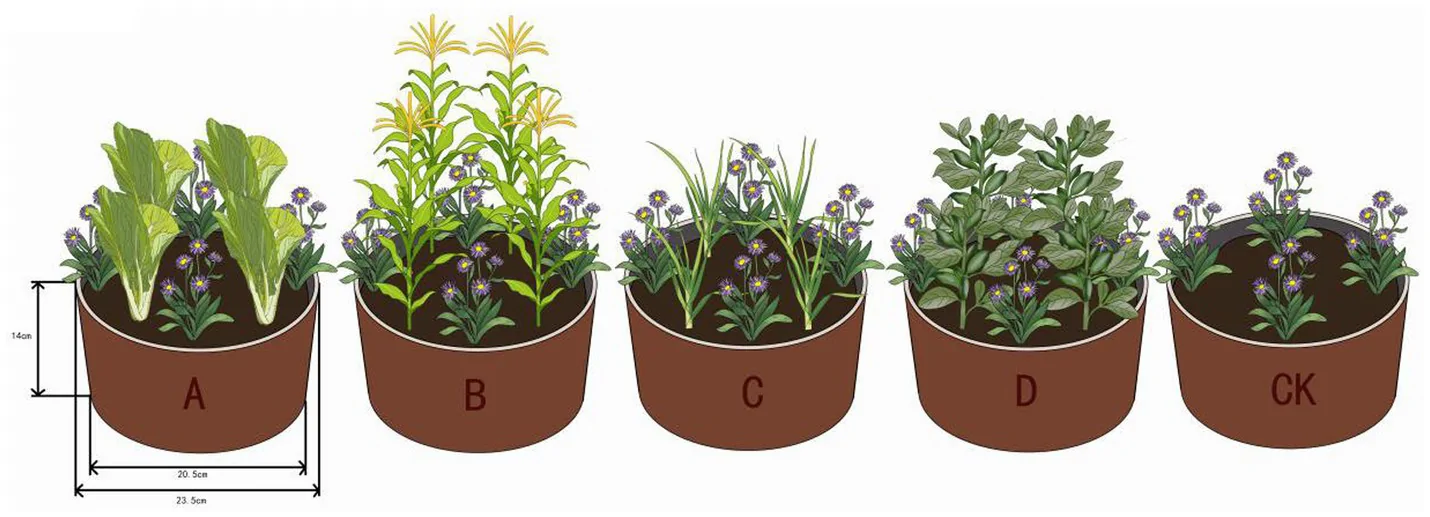
Layout of the pot cultivation. CK, *Erigeron breviscapus* by monoculture; A, *Brassica juncea* L. companion cultivation; B, corn companion cultivation; C, garlic companion cultivation; D, broad bean cultivation.

According to the method described by Gao and Zhang (2023), the experiment was conducted in the greenhouse of Honghe University, Mengzi County, Yunnan Province, China. Soil samples were collected from the rhizosphere of *E. breviscapus* plants that had been continuously cropped for 3 years in Luxi County. Five treatments were established: *E. breviscapus* monoculture (control, CK), (A) intercropping with *B. juncea* L., (B) intercropping with corn, (C) intercropping with purple garlic, and (D) intercropping with broad bean (Figure 1). Each treatment had three replicate pots, with each replicate containing three pots, resulting in a total of 45 pots arranged randomly. Depending on the treatment, fertilizer was mixed uniformly with 2.0 kg of soil and placed in pots (20.5 cm in diameter and 14 cm in height). After sowing the seeds, the pots were temporarily covered with a white plastic film. *E. breviscapus* was sown on 18 December 2022, and seedling transplanting was performed on 12 January 2023. Before planting, 8 g of compound fertilizer (N:P:K = 15:15:15) was applied per pot as the base fertilizer. Thirty days later, 2 g of compound fertilizer (N:P:K = 15:15:15) was top-dressed per pot. At 120 days, rhizosphere soil samples of *E. breviscapus* were collected simultaneously and divided into two portions: one was stored at 4 °C and the other at −80 °C for the determination of soil enzyme activities and soil DNA extraction, respectively. Deep sequencing of bacterial and fungal samples was performed on the MiSeq platform by Biomarker Technologies Co., Ltd. (Beijing, China).

### 2.3 Determination growth and quality parameters of *Erigeron breviscapus*

According to the method described by Martin et al. (2025), *E. breviscapus* plants were sampled at 60 days after planting, and subsequent samples were collected every 20 days.

#### 2.3.1 Determination of total flavonoid content

According to the method described by Pawel et al. (2021), 0.5 g of *E. breviscapus* leaves was weighed, and 10 mL of 70% ethanol was added, followed by the ultrasonic treatment for 20 min. Thereafter, the solution was centrifuged at 10,000 rpm for 2 min at 25 °C, and the supernatant was collected for analysis. Subsequently, 1 mL of the extract was mixed with 1 mL of ethanol, followed by the addition of 0.3 mL of 5% sodium nitrite, and the mixture was allowed to stand for 6 min. After 6 min, 0.3 mL of 10% aluminum chloride solution was added, and the mixture was allowed to stand for another 6 min, followed by the addition of 0.3 mL of 4% sodium hydroxide. The solution was mixed thoroughly and then allowed to stand for 10 min. The absorbance was measured at 510 nm. The experiment was performed in triplicate, and catechin standards were used to generate a calibration curve.

#### 2.3.2 Determination of scutellarin content

According to the method described by Bai et al. (2020), the preparation of the reference substance was prepared as follows: A dried scutellarin reference standard was accurately weighed and dissolved in methanol to prepare a standard solution with a concentration of 0.1 mg/mL. Regarding the preparation of the test solution: A 1.0-g portion of *E. breviscapus* powder was transferred into a 150-ml conical flask, to which 100 mL of 50% methanol was added accurately. Ultrasonic extraction was performed for a duration of 1 h, after which the mixture was left to cool. Any weight loss was compensated for by adding 50% methanol, and the mixture was thoroughly shaken afterwards. The resulting liquid was passed through a 0.45-μm microporous membrane filter, and the collected filtrate was used as the test solution.

Details of the chromatographic parameters were listed as follows: Octadecylsilane-bonded silica gel was used as the stationary phase, while a mixture of methanol and 0.1% phosphoric acid at a volumetric ratio of 40:60 served as the mobile phase. The flow rate was maintained at 1.0 mL/min, and detection was conducted at a wavelength of 335 nm. For the quantitative analysis of sample content, separate aliquots ranging from 5 to 10 μL of both the reference standard solution and the test solution were injected into the liquid chromatograph. Subsequent measurement was performed, and final results were computed in accordance using the designated calculation formula: *C* = (*Cs*×*A*1 × 100 mL) / (*A*2 × 1,000 mg) × 100, where *C* is the scutellarin content, *Cs* is the concentration of the reference substance solution (mg/ml), *A*1 is the integral of the sample peak area, and *A*2 is the integral of the reference substance peak area.

#### 2.3.3 Measurement of morphological indicators of above ground parts

According to the method described by Gai et al. (2025), plant height, stem diameter, leaf length, leaf width, and leaf number of *E. breviscapus* were measured 60 days after planting. Subsequently, measurements were taken every 20 days.

#### 2.3.4 Measurement of morphological indicators of underground parts

As described by Li et al. (2025), soil adhering to the roots of *E. breviscapus* was removed. The cleaned roots were scanned with a root scanner (LK-1910), and finally, root analysis software (WinRHIZO 2008a) was used to obtain the morphological characteristic indicators of the root system. The recorded data included total root length, total root surface area, average root diameter, total root volume, specific root length, total root number, specific surface area, and specific tissue density.

### 2.4 Determination of soil enzyme activity, nutrients, and microbial community

According to the method described by Xia et al. (2024), soil samples were collected from the rhizosphere of *E. breviscapus*. The sampling times included the early stage (12 January 2023), the middle stage (24 March 2023), and the late stage (15 May 2023). The soil samples were divided into two parts: one part was preserved at low temperature for the determination of soil nutrients and enzyme activity, and the other was stored in an ice box, transported to the laboratory, and then stored at −80 °C for the determination of soil microbial community structure. Soil samples from the late stage were used for microbial community determination.

### 2.5 Statistical analysis and data quality control

Microsoft Excel 2016 and GraphPad Prism were adopted for raw data sorting and visualization. A one-way analysis of variance (ANOVA), followed by Tukey’s HSD (honestly significant difference) multiple comparison test (*p* < 0.05), was used to compare differences of agronomic traits, soil physicochemical properties, enzyme activities and microbial alpha diversity among treatments. Prior to all statistical inference, rigorous assumption testing was conducted to guarantee methodological validity. For agronomic morphological indexes, soil physicochemical properties, enzyme activities and microbial alpha diversity indices, the Shapiro–Wilk normality test, and Levene homogeneity of variance test were performed. All datasets satisfied normal distribution (*p* > 0.05) and homogeneous variance (*p* > 0.05); therefore, ANOVA followed by Tukey’s HSD post-hoc multiple comparison test at a *p-*value of < 0.05 was utilized to distinguish inter-treatment differences. Tukey’s HSD was chosen for its strict control of cumulative Type I error in multiple pairwise comparisons. Spearman’s rank correlation analysis was applied to quantify the associations between rhizosphere microbial relative abundance and soil environmental variables. The Shapiro–Wilk tests indicated that sequencing-based microbial abundance data did not meet the bivariate normality requirement of parametric Pearson correlation. The non-parametric Spearman’s correlation method, based on rank order rather than raw values, is suitable for skewed microbiome datasets and has become the conventional analytical tool in rhizosphere microecology studies. Correlations with a *p-*value of <0.05 were considered statistically significant.

## 3 Results

### 3.1 Impact of intercropping with different crops on the total flavonoid content of *Erigeron breviscapus*

As indicated in Table 1, with the extension of planting time, the total flavonoid content of treatments CK (control), A, C, and D exhibited a trend of an initial increase followed by a gradual decrease. Specifically, treatment B showed increases of 222.45, 16.39, and 23.39% compared with the control at 60, 100, and 120 days, respectively. Treatment D showed increases of 15.83, 7.30, and 6.82% compared with the control at 60, 80, and 100 days, respectively.

**Table 1:** Effects of companion cropping on the total flavonoids content of *Erigeron breviscapus*.

Treatment	60 d	80 d	100 d	120 d
CK	4.23 ± 0.87 b	13.01 ± 2.85 a	9.82 ± 2.67 a	7.31 ± 1.06 ab
A	10.93 ± 4.17 a	5.16 ± 0.69 b	9.58 ± 0.55 a	5.71 ± 0.77 ab
B	13.64 ± 1.73 a	6.32 ± 0.47 b	11.43 ± 0.94 a	9.02 ± 3.35 a
C	4.06 ± 0.74 b	11.49 ± 1.80 a	8.81 ± 1.94 a	4.67 ± 1.03 b
D	4.90 ± 1.63 b	13.96 ± 2.59 a	10.49 ± 2.61 a	6.16 ± 1.49 ab

Values are the mean ± standard deviation (SD) of three replicates. Different letters in the same column indicate significant differences (*p* < 0.05).

### 3.2 Impact of intercropping with different crops on the content scutellarin of *Erigeron breviscapus*

The scutellarin content of *E. breviscapus* is shown in Table 2. Compared with the control, the scutellarin content of treatments A, B, C, and D was not significantly different, and the scutellarin content of all treatments was significantly lower than that of the control and decreases by 31.73, 31.14, 25.75, and 29.94%, respectively, at 120 days. With increasing planting time, the control, C, and D treatments showed a trend of an initial increase, followed by decrease and then a subsequent increase, whereas treatments A and B showed a trend of an initial decrease followed by an increase. At 60 days, there were no significant differences among the four treatments, but treatments B and D showed increases in scutellarin content by 6.29 and 0.78% compared with the control, respectively. At 80 days, the scutellarin content of treatments A and B was significantly lower than that of the control by 52.83 and 69.39%, respectively. At 100 days, there was a significant difference between treatment A and the other treatments, with the scutellarin content of treatment A being 54.26% lower than that of the control.

**Table 2:** Effects of companion cropping on the scutellarin content of *Erigeron breviscapus*.

Treatment	60 d	80 d	100 d	120 d
CK	1.27 ± 0.40 a	1.83 ± 0.22 a	1.25 ± 0.27 a	1.67 ± 0.12 a
A	1.06 ± 0.01 a	0.79 ± 0.06 b	0.59 ± 0.19 b	1.14 ± 0.01 b
B	1.35 ± 0.30 a	0.56 ± 0.12 b	1.17 ± 0.01 a	1.15 ± 0.02 b
C	0.98 ± 0.06 a	1.38 ± 0.04 a	1.14 ± 0.14 a	1.24 ± 0.04 b
D	1.28 ± 0.00 a	1.76 ± 0.38 a	1.20 ± 0.10 a	1.17 ± 0.11 b

Values are the mean ± standard deviation (SD) of three replicates. Different letters in the same column indicate significant differences (*p* < 0.05).

### 3.3 Impact of intercropping with different crops on the growth parameters of *Erigeron breviscapus*

#### 3.3.1 Impact of intercropping with different crops on the morphological indicators of the aboveground part of *Erigeron breviscapus*

According to Table 3, intercropping led to a decrease in leaf length, leaf width, and leaf number of *E. breviscapus* but resulted in an increase in plant height for treatments A, B, and D. Plant height and leaf number fluctuated with the extension of planting time. At 60 and 80 days, the plant height of treatment A was significantly higher than that of CK, increasing by 31.38 and 40.50%, respectively. At 100 days, the plant height of treatment C was significantly lower than that of CK, with a decrease of 19.49%, and the leaf number of treatment C was significantly reduced by 45.90% compared with CK. At 120 days, the leaf number of treatments A, B, and C was significantly decreased by 33.65, 35.64, and 25.00% compared with CK, respectively. Leaf width showed no significant change throughout the planting period, whereas leaf length remained stable in the early stage but gradually decreased after an initial increase during the later stage. At 100 days, the leaf length of treatments A and B was significantly lower than that of CK, with decreases of 20.58 and 2.64%, respectively. At 120 days, treatment D showed a reduction in leaf length of 22.79% compared with CK. In summary, monoculture of *E. breviscapus* is more beneficial for the growth of its aboveground parts compared with intercropping.

**Table 3:** Effects of companion cropping on the above ground growth of *Erigeron breviscapus*.

Day	Treatment	Plant height/cm	Leaf length/cm	Leaf width/cm	Leaf number/count
60 d	CK	34.54 ± 14.65 a	9.94 ± 1.93 a	0.58 ± 0.19 a	12.78 ± 3.67 a
	A	45.38 ± 11.57 a	11.13 ± 2.02 a	1.94 ± 0.40 a	13.44 ± 3.40 a
	B	40.47 ± 9.34 a	11.04 ± 1.97 a	1.92 ± 0.42 a	12.33 ± 2.74 a
	C	40.12 ± 4.36 a	10.54 ± 2.52 a	2.40 ± 2.14 a	15.00 ± 3.32 a
	D	42.39 ± 9.11 a	9.87 ± 2.08 a	1.81 ± 0.34 a	13.89 ± 5.56 a
80 d	CK	30.81 ± 9.83 b	12.04 ± 3.15 a	1.46 ± 0.12 a	15.33 ± 5.55 a
	A	43.29 ± 9.69 a	11.33 ± 2.58 a	2.28 ± 2.28 a	17.00 ± 4.33 a
	B	39.26 ± 6.30 a	14.50 ± 3.99 a	1.69 ± 0.46 a	16.78 ± 5.30 a
	C	40.17 ± 4.22 a	13.83 ± 3.99 a	1.60 ± 0.47 a	16.78 ± 3.15 a
	D	37.33 ± 6.00 ab	11.18 ± 4.52 a	1.54 ± 0.29 a	18.33 ± 5.79 a
100 d	CK	24.01 ± 3.26 a	20.02 ± 3.58 a	2.71 ± 0.88 a	12.33 ± 4.24 a
	A	16.86 ± 3.62 c	15.90 ± 3.55 c	3.28 ± 4.78 a	12.67 ± 6.40 a
	B	20.43 ± 2.30 b	19.49 ± 3.01 b	1.96 ± 0.46 a	10.89 ± 2.67 a
	C	19.33 ± 3.30 bc	18.66 ± 3.10 bc	2.17 ± 0.34 a	6.67 ± 1.94 b
	D	18.57 ± 3.81 bc	17.77 ± 3.57 bc	2.12 ± 0.58 a	11.67 ± 3.97 a
120 d	CK	21.92 ± 4.31 a	21.32 ± 4.31 a	2.74 ± 0.84 a	11.56 ± 3.87 ab
	A	18.92 ± 2.27 ab	18.37 ± 1.85 ab	1.99 ± 0.35 b	7.67 ± 2.65 c
	B	18.51 ± 3.50 ab	18.04 ± 3.31 ab	1.87 ± 0.42 b	7.44 ± 2.19 c
	C	18.71 ± 3.84 ab	17.93 ± 3.90 ab	2.33 ± 0.48 ab	8.67 ± 3.43 bc
	D	16.99 ± 4.90 b	16.46 ± 4.64 b	1.77 ± 0.57 b	14.44 ± 5.08 a

Values are the mean ± standard deviation (SD) of three replicates. Different letters in the same column indicate significant differences (*p* < 0.05).

#### 3.3.2 Impact of intercropping with different crops on the morphological indicators of the underground part of *Erigeron breviscapus*

As shown in Table 4, although there were no significant differences in average root diameter and specific surface area among treatments A, B, C, and D, both indicators were significantly lower than those of the control. No significant differences in specific tissue density were observed between treatments A, B, C, D, and the control; however, the specific tissue density of all four treatments was higher than that of the control. Based on these results, monoculture of *E. breviscapus* was more beneficial for its root growth.

**Table 4:** Effects of companion cropping on the root morphology of *Erigeron breviscapus*.

Treatment	Root Length (cm)	Total root surface area (cm^2^)	Average root diameter (mm)	Total root volume (cm^3^)	Specific root length (cm/g)	Specific surface area (cm^2^/g)	Specific tissue density (g/cm^3^)
CK	17.77 ± 4.70 a	13.69 ± 3.81 a	2.45 ± 0.28 a	0.85 ± 0.26 a	194.61 ± 42.64 a	151.98 ± 42.70 a	0.11 ± 0.06 a
A	8.34 ± 5.06 a	3.63 ± 2.52 a	1.43 ± 0.29 b	0.13 ± 0.10 a	194.56 ± 55.88 a	85.55 ± 26.11 b	0.39 ± 0.20 a
B	12.17 ± 4.71 a	6.31 ± 2.98 a	1.61 ± 0.27 b	0.27 ± 0.16 a	192.12 ± 75.45 a	97.21 ± 39.12 b	0.53 ± 0.77 a
C	14.16 ± 2.98 a	7.47 ± 2.11 a	1.68 ± 0.31 b	0.33 ± 0.15 a	203.83 ± 13.77 a	107.44 ± 20.33 b	0.25 ± 0.09 a
D	13.79 ± 2.87 a	7.45 ± 1.56 a	1.73 ± 0.15 b	0.32 ± 0.08 a	171.53 ± 15.92 a	92.55 ± 7.66 b	0.25 ± 0.03 a

Values are the mean ± standard deviation (SD) of three replicates. Different letters in the same column indicate significant differences (*p* < 0.05).

### 3.4 Restoration effect of intercropping of different crops on soil nutrients

As shown in Supplementary Table S1 and Table 5, the soil pH showed a trend of an initial decrease and then an increase. The pH of treatment C increased by 1.07 and 1.02% relative to CK in March and May, respectively, while the pH of treatment D increased by 1.68 and 1.15% relative to CK in March and May, respectively. Compared to other treatments, treatment C had a better effect on improving alkaline hydrolyzable nitrogen, and treatment D had a better effect on improving available potassium.

**Table 5:** Remediation of soil nutrients for *Erigeron breviscapus* by different companion cropping.

Year	Treatment	pH	Organic matter (g/kg)	Available potassium (mg/kg)	Available phosphorus (mg/kg)	Alkali-hydrolyzable nitrogen (mg/kg)	Total potassium (g/kg)	Total phosphorus (g/kg)	Total nitrogen (g/kg)
2023.03	CK	6.54 ± 0.01 a	16.69 ± 0.34 b	538.31 ± 37.78 a	4.41 ± 1.58a	5.60 ± 1.14 ab	13.08 ± 0.35 b	0.28 ± 0.03 b	2.11 ± 0.06 a
	A	6.17 ± 0.19 b	17.59 ± 0.34 ab	472.43 ± 7.69 b	6.51 ± 1.73 a	5.60 ± 1.14 ab	15.06 ± 0.15 a	0.32 ± 0.02 a	1.29 ± 0.77 a
	B	6.47 ± 0.08 a	18.17 ± 0.20 a	495.31 ± 8.31 ab	6.30 ± 3.63 a	6.53 ± 1.32 a	13.08 ± 0.76 b	0.32 ± 0.01 a	1.43 ± 0.90 a
	C	6.61 ± 0.03 a	14.71 ± 0.96 c	500.57 ± 11.75 ab	11.08 ± 6.00 a	3.27 ± 1.32 b	13.26 ± 0.72 b	0.27 ± 0.01 b	1.49 ± 0.82 a
	D	6.65 ± 0.01 a	11.98 ± 0.55 d	546.35 ± 32.22 a	7.65 ± 3.91 a	7.00 ± 1.14 a	12.71 ± 0.09 b	0.31 ± 0.01 a	2.08 ± 0.18 a
2023.05	CK	6.86 ± 0.01 b	26.66 ± 3.37 b	528.72 ± 6.12 a	7.78 ± 2.05 ab	56.93 ± 29.75 a	13.20 ± 0.76 ab	0.26 ± 0.03 c	2.14 ± 0.13 a
	A	6.91 ± 0.02 ab	31.15 ± 1.17 a	432.84 ± 7.96 c	11.20 ± 1.15 ab	8.87 ± 3.49 a	12.83 ± 0.15 b	0.34 ± 0.01 a	1.93 ± 0.14 a
	B	7.04 ± 0.08 a	24.19 ± 0.37 bc	391.70 ± 8.19 d	8.98 ± 2.54 a	34.53 ± 35.47 a	13.94 ± 0.15 a	0.31 ± 0.01 b	1.96 ± 0.11 a
	C	6.93 ± 0.10 ab	22.53 ± 1.16 c	508.00 ± 6.87 b	6.75 ± 0.62 b	60.67 ± 11.10 a	11.59 ± 0.53 c	0.33 ± 0.02 ab	1.97 ± 0.13 a
	D	6.94 ± 0.01 ab	23.20 ± 0.44 bc	533.67 ± 3.47 a	7.64 ± 0.93 ab	46.20 ± 41.59 a	12.46 ± 0.40 bc	0.32 ± 0.03 ab	2.12 ± 0.17 a

Values are the mean ± standard deviation (SD) of three replicates. Different letters in the same column indicate significant differences (*p* < 0.05).

### 3.5 Restoration effect of intercropping of different crops on soil enzyme activity

As shown in Supplementary Table S2 and Table 6, the catalase activity under different treatments showed a trend of an initial decrease and then an increase from January to May. The invertase activity increased continuously, while the acid phosphatase and urease activities decreased continuously. Compared with the control, treatment B significantly enhanced soil urease activity. The catalase activity in treatment C was significantly higher than that in CK, with specific increases of 3.21 and 9.27% in March and May, respectively. The acid phosphatase activity in treatment B increased by 16.67 and 10.34% compared with the control in March and May, respectively, while treatment A increased by 8.33 and 1.72% compared with the control in March and May, respectively.

**Table 6:** Remediation of soil enzyme activity in *Erigeron breviscapus* by different companion cropping.

Year	Treatment	Sucrase activity [mg/(g·24 h)]	Urease activity [mg/(g·24 h)]	Catalase activity (mL/g)	Acid phosphatase activity [mg/(g·24 h)]
2023.03	CK	19.04 ± 0.14ab	0.08 ± 0.02b	2.49 ± 0.14ab	0.48 ± 0.06c
	A	18.38 ± 0.38b	0.05 ± 0.04b	1.73 ± 0.41c	0.52 ± 0.04c
	B	18.81 ± 0.20ab	0.29 ± 0.37a	2.19 ± 0.09abc	0.56 ± 0.01bc
	C	19.63 ± 0.86a	0.10 ± 0.06b	2.57 ± 0.05a	0.80 ± 0.06a
	D	19.12 ± 0.07ab	0.11 ± 0.07b	2.03 ± 0.05bc	0.76 ± 0.01ab
2023.05	CK	19.10 ± 0.17a	0.04 ± 0.03b	2.91 ± 0.14a	0.58 ± 0.04a
	A	18.60 ± 0.16b	0.06 ± 0.03b	2.99 ± 0.47a	0.59 ± 0.03a
	B	18.93 ± 0.11a	0.13 ± 0.11a	3.26 ± 0.30a	0.64 ± 0.01a
	C	19.07 ± 0.18a	0.08 ± 0.05ab	3.18 ± 0.09a	0.33 ± 0.01b
	D	19.11 ± 0.06a	0.08 ± 0.03ab	3.14 ± 1.17a	0.55 ± 0.01a

Values are the mean ± standard deviation (SD) of three replicates. Different letters in the same column indicate significant differences (*p* < 0.05).

### 3.6 Impact of intercropping with different crops on the fungi in the rhizosphere soil of *Erigeron breviscapus*

#### 3.6.1 Impact of intercropping with different crops on the fungal community in the rhizosphere soil of *Erigeron breviscapus*

**Figure 2:**
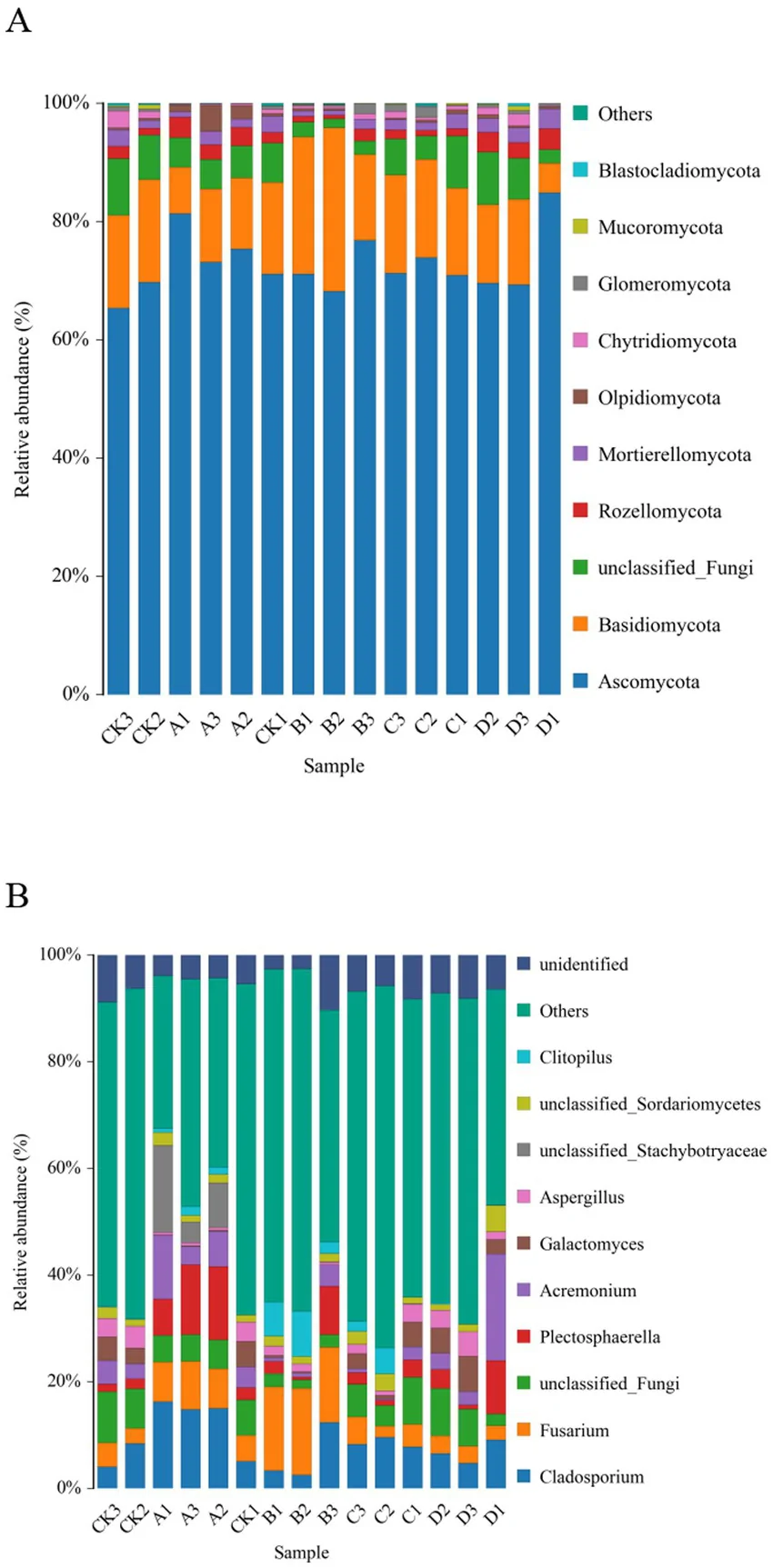
The distribution of dominant fungi at phyla level **(A)** and genus level **(B)** of *Erigeron breviscapus* by companion cropping.

**Figure 3:**
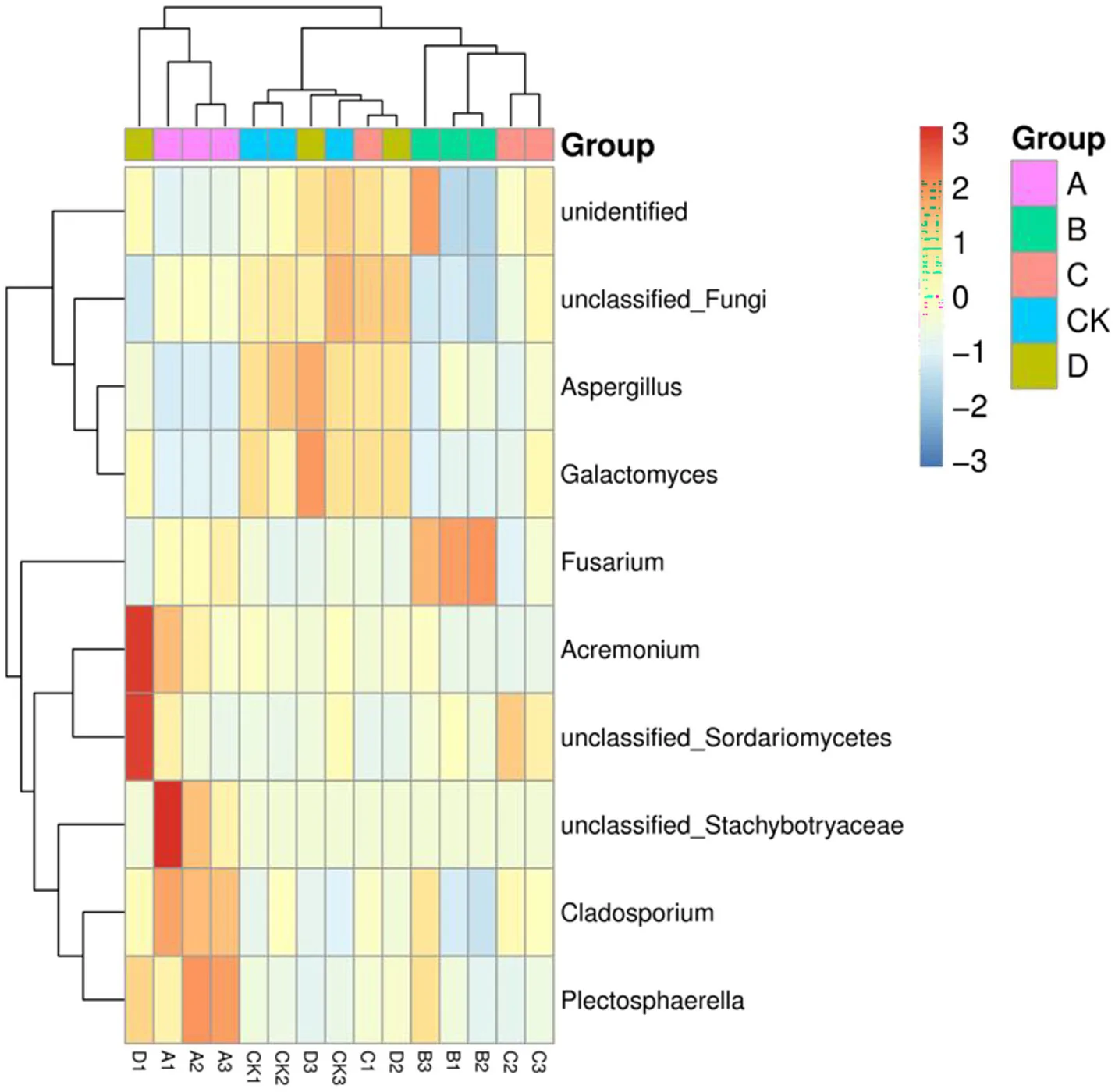
The distribution of dominant fungi at genus level of *Erigeron breviscapus* by companion cropping.

*Ascomycota* and *Basidiomycota* dominated the fungal communities across all treatments, with their relative abundances ranging from 36.65 to 72.80% and from 9.23 to 45.73%, respectively (Figure 2A). Intercropping types clearly reshaped the fungal taxonomic composition under 3-year continuous cropping soil. The relative abundance of *Ascomycota* followed the order A > D > C > B > CK. At the genus level, distinct dominant fungal genera were observed among the different treatments (Figure 2B). Treatment B showed the highest relative abundance of pathogenic *Fusarium*, while *Cladosporium* dominated in treatment A. The genus-level cluster heatmap further revealed community similarity: treatments B and C shared highly consistent fungal assembly patterns and clustered into one group (Figure 3). By contrast, the fungal community of treatment A showed a distinct structural separation from other groups with low similarity to the remaining treatments.

#### 3.6.2 Operational taxonomic unit analysis of fungi in the rhizosphere soil of *Erigeron breviscapus* under different crops intercropping

**Figure 4:**
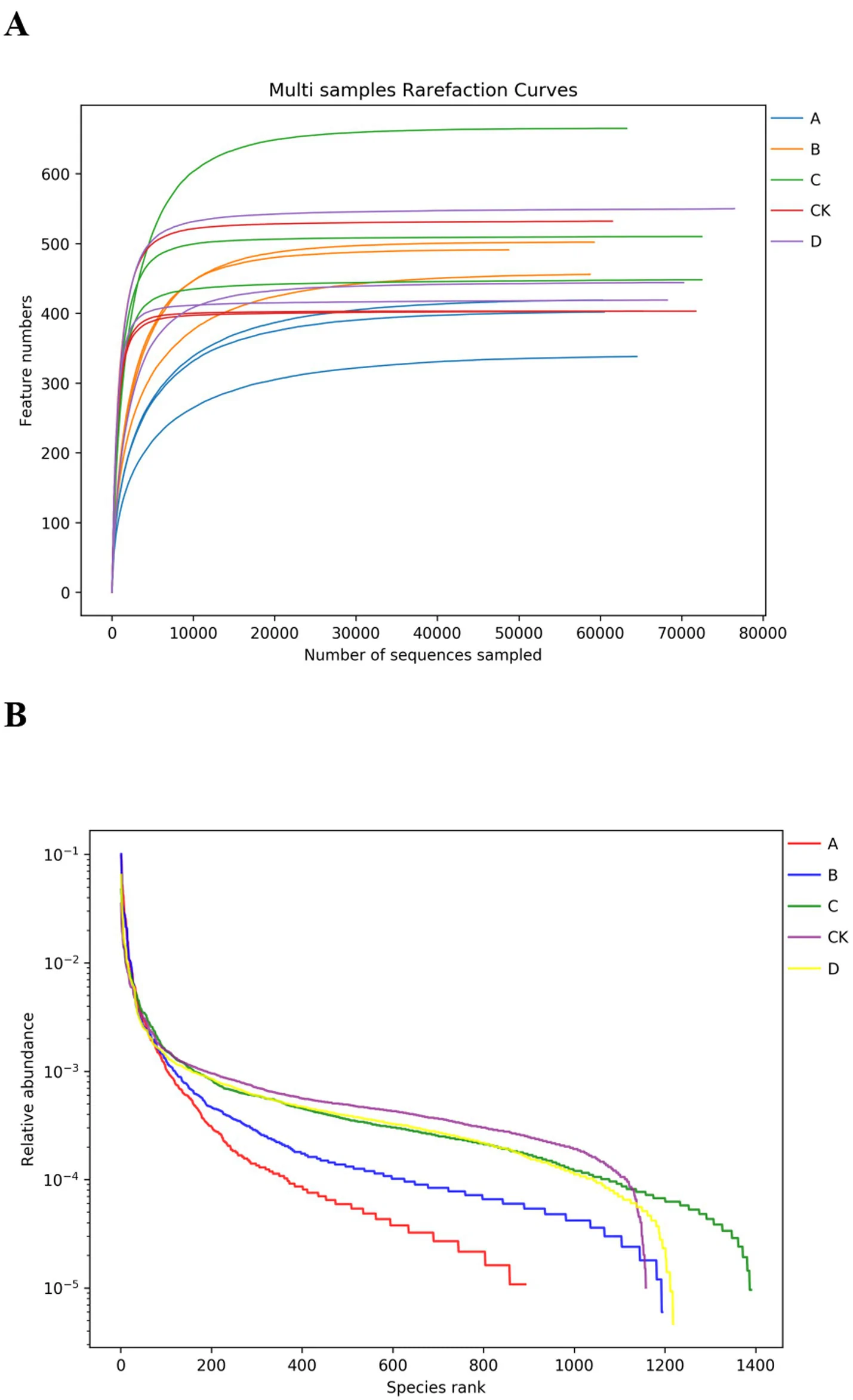
Rarefaction curves **(A)** and species rank abundance curves **(B)** for fungi of *Erigeron breviscapus* by companion cropping.

**Figure 5:**
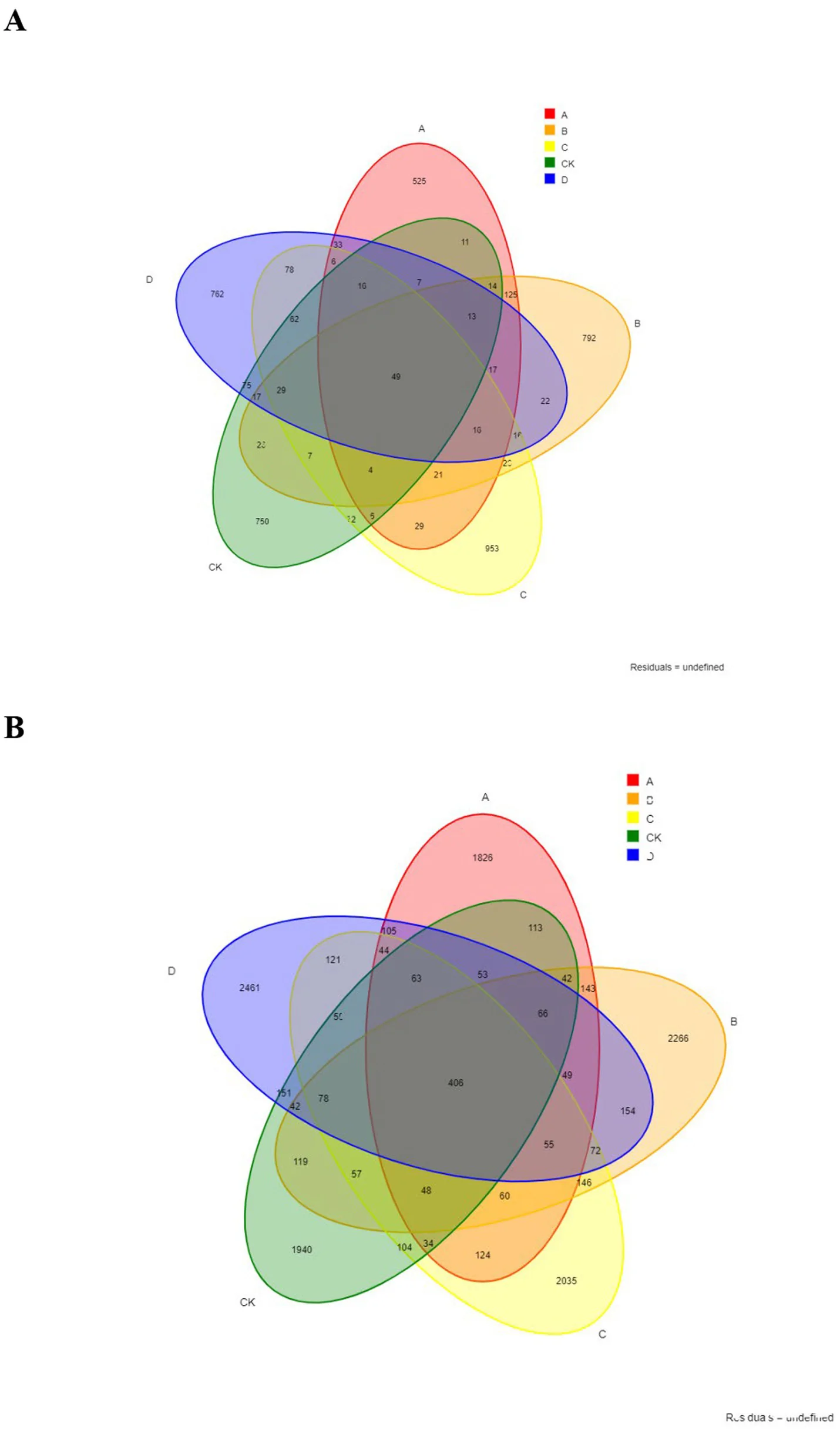
Venn diagram of fungi **(A)** and bacteria **(B)** of *Erigeron breviscapus* by companion cropping.

The dilution curves of the five fungal treatments showed a gradual flattening trend, indicating that the sequencing depth covered the vast majority of fungal types in the samples (Figure 4A). This suggests that the sequencing quantity is reasonable and can be used to study the community structure of the fungal population. From the rank-abundance curve, it can be seen that the fungal abundance was ranked in the order C > D > B > CK > A (Figure 4B). The Venn diagram of operational taxonomic units (OTUs) under different treatments showed that the total fungal OTUs for CK, A, B, C, and D were 1,158, 886, 1,168, 1,302, and 1,218, respectively. The number of unique OTUs for CK, A, B, C, and D was 750, 525, 792, 953, and 762, respectively, with the remaining OTUs shared among the treatments. Except for the treatment intercropped with *B. juncea* L., the number of unique fungal OTUs in all other intercropping treatments was higher than that in the *E. breviscapus* monoculture (Figure 5A).

#### 3.6.3 Analysis of fungal diversity in the rhizosphere soil of *Erigeron breviscapus* under different crops intercropping

**Figure 6:**
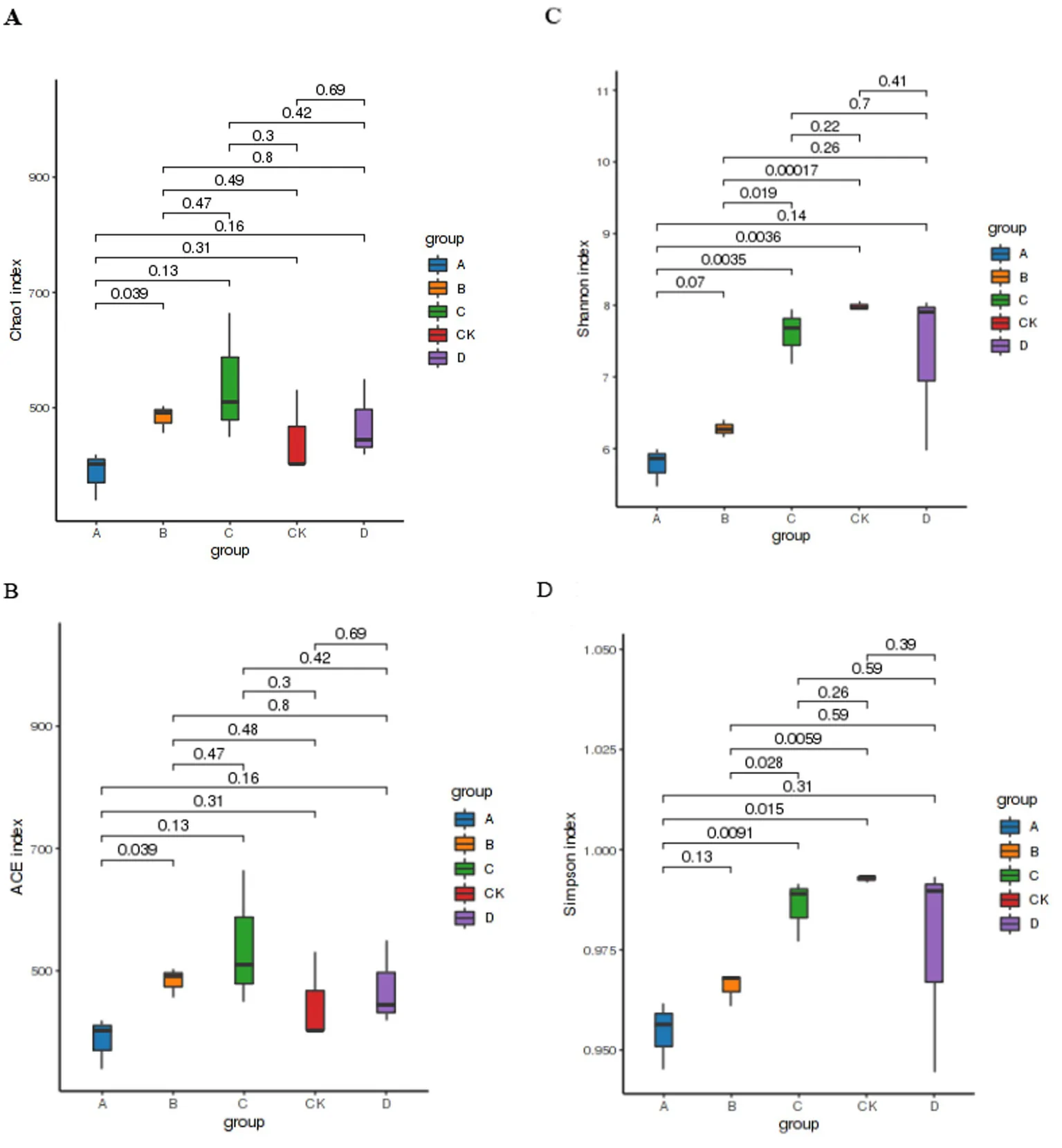
Alpha diversity of rhizosphere fungal communities of *Erigeron breviscapus* by companion cropping The numeric values above each boxplot indicate adjusted *P*-values for pairwise comparisons across experimental groups.

The fungal communities associated with different treatments of *E. breviscapus* were analyzed for alpha diversity. The ACE and Chao1 indices primarily measure species richness, while the Shannon and Simpson indices measure both richness and evenness; the higher values indicate greater diversity. As shown in Figures 6A,B, significant differences in both the Chao1 and ACE indices were observed between treatments A and B. When the treatments were compared in terms of the Shannon and Simpson indices, significant differences were only observed between treatment A and treatment C, as well as between treatment A and CK (Figures 6C,D). These findings suggest that the fungal abundance of the treatment intercropped with corn was higher than that of the treatment intercropped with *B. juncea* L. and that the fungal diversity of the treatment intercropped with *B. juncea* L. was different from and higher than that of the *E. breviscapus* monoculture and the treatment intercropped with garlic.

### 3.7 Impact of intercropping with different crops on the bacteria in the rhizosphere soil of *Erigeron breviscapus*

#### 3.7.1 Impact of intercropping with different crops on the bacterial community in the rhizosphere soil of *Erigeron breviscapus*

**Figure 7:**
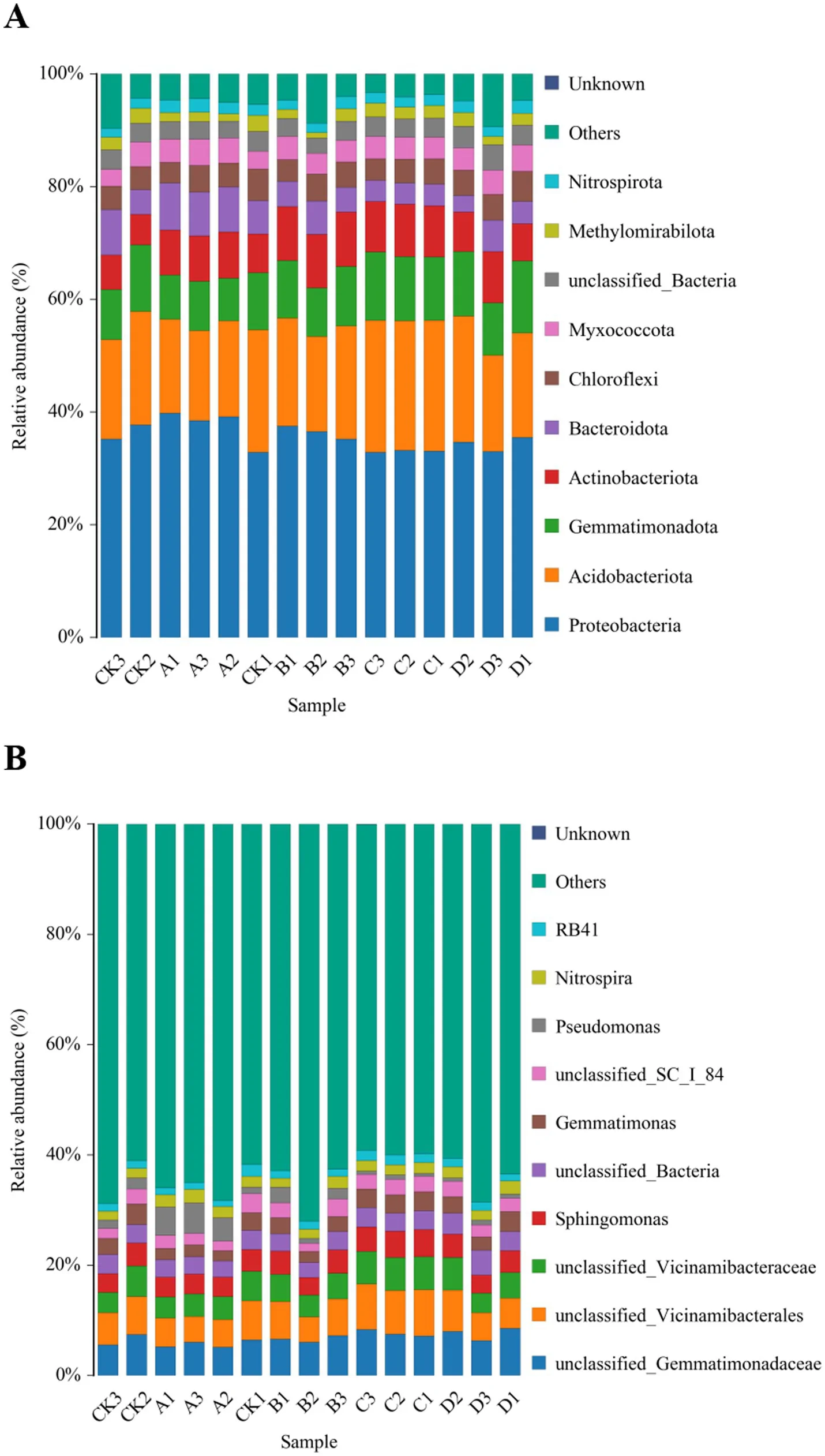
The distribution of dominant bacteria at phyla level **(A)** and genus level **(B)** of *Erigeron breviscapus* by companion cropping.

**Figure 8:**
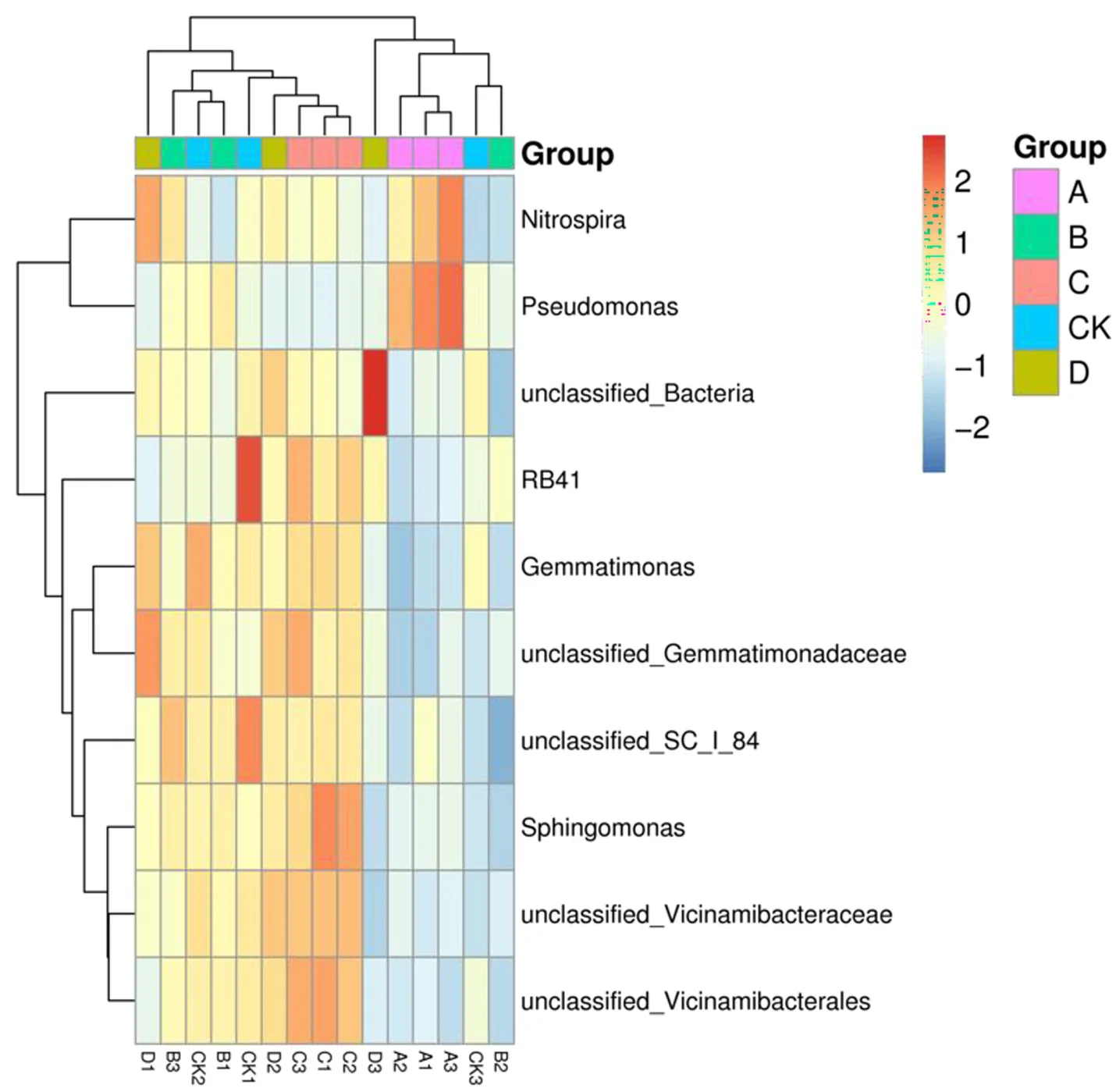
The distribution of dominant bacteria at genus level of *Erigeron breviscapus* by companion cropping.

A total of 1,228 bacterial species belonging to 34 phyla, 94 classes, 260 orders, 517 families, and 990 genera were identified from 15 soil samples using high-throughput sequencing and analysis. *Proteobacteria* (32.88–39.77%), *Acidobacteriota* (15.92–23.35%), and *Gemmatimonadota* (7.57–12.72%) were the dominant phyla present across all treatments (Figure 7A), respectively. Both *Actinobacteriota* and *Proteobacteria* reached their highest abundances in treatment B. The abundance of *Proteobacteria* was the highest in treatment A, followed by treatments in the order B > CK > D > C, while treatment C had the highest abundance of *Acidobacteriota*, followed by treatments in the order CK > D > B > A. With respect to the dominant genera, *Bacillus* was the dominant genus in treatment A (Figure 7B), while *Vicinamibacter* was the dominant genus in treatment C. To better assess the distribution and community composition of these endophytic bacterial genera, the top 10 bacterial genera with the highest relative abundances were selected, and a species distribution heatmap was generated for cluster analysis (Figure 8). According to the analysis, at the bacterial genus level, samples from treatments B and CK were clustered into the same group and exhibited similar community structure characteristics. In contrast, samples from treatment A exhibited a distinct community structure at the genus level, which was significantly different from that of the other samples.

#### 3.7.2 OTU analysis of bacteria in the rhizosphere soil of *Erigeron breviscapus* under different crops intercropping

**Figure 9:**
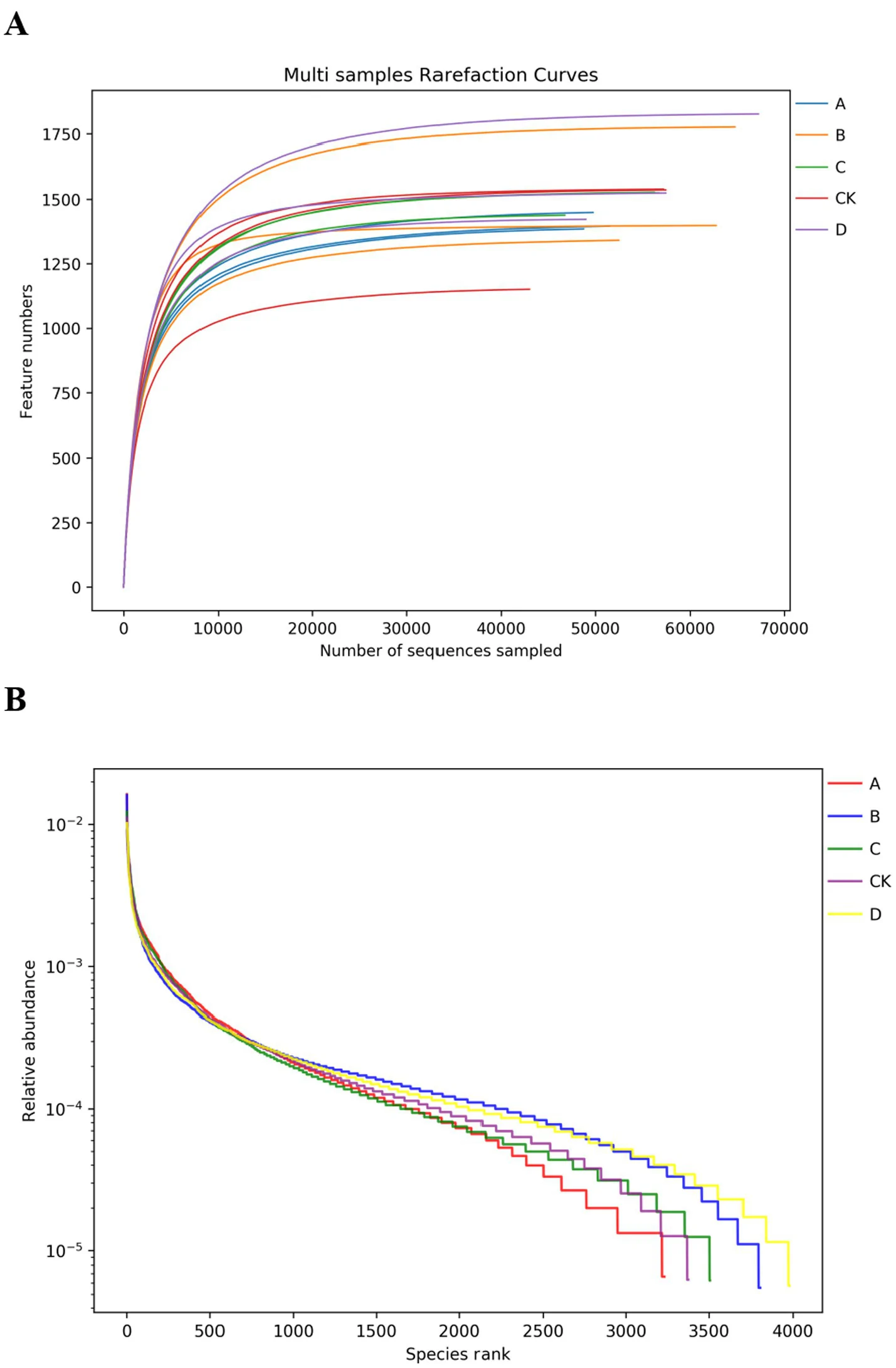
Rarefaction curves **(A)** and species rank abundance curves **(B)** for bacteria of *Erigeron breviscapusin* by companion cropping.

The total bacterial OTUs for CK, A, B, C, and D were 3,375, 3,197, 3,946, 3,458, and 3,979, respectively. The number of unique OTUs specific to CK, A, B, C, and D was 1940, 1826, 2,266, 2035, and 2,461, respectively. Except for the treatment intercropped with *B. juncea* L., the number of unique bacterial OTUs in all other intercropping treatments was higher than that in the *E. breviscapus* monoculture (Figure 5B). The dilution curves of the bacteria in each treatment (Figure 9A) showed a gradual flattening trend, indicating that the sequencing depth covered the vast majority of bacterial types in the samples. These findings suggest that the sequencing quantity is reasonable and can be used to study the community structure of the bacterial community. From the abundance chart (Figure 9B), it can be seen that the order of bacterial abundance was D > B > C > CK > A.

#### 3.7.3 Analysis of bacteria diversity in the rhizosphere soil of *Erigeron breviscapus* under different crops intercropping

**Figure 10:**
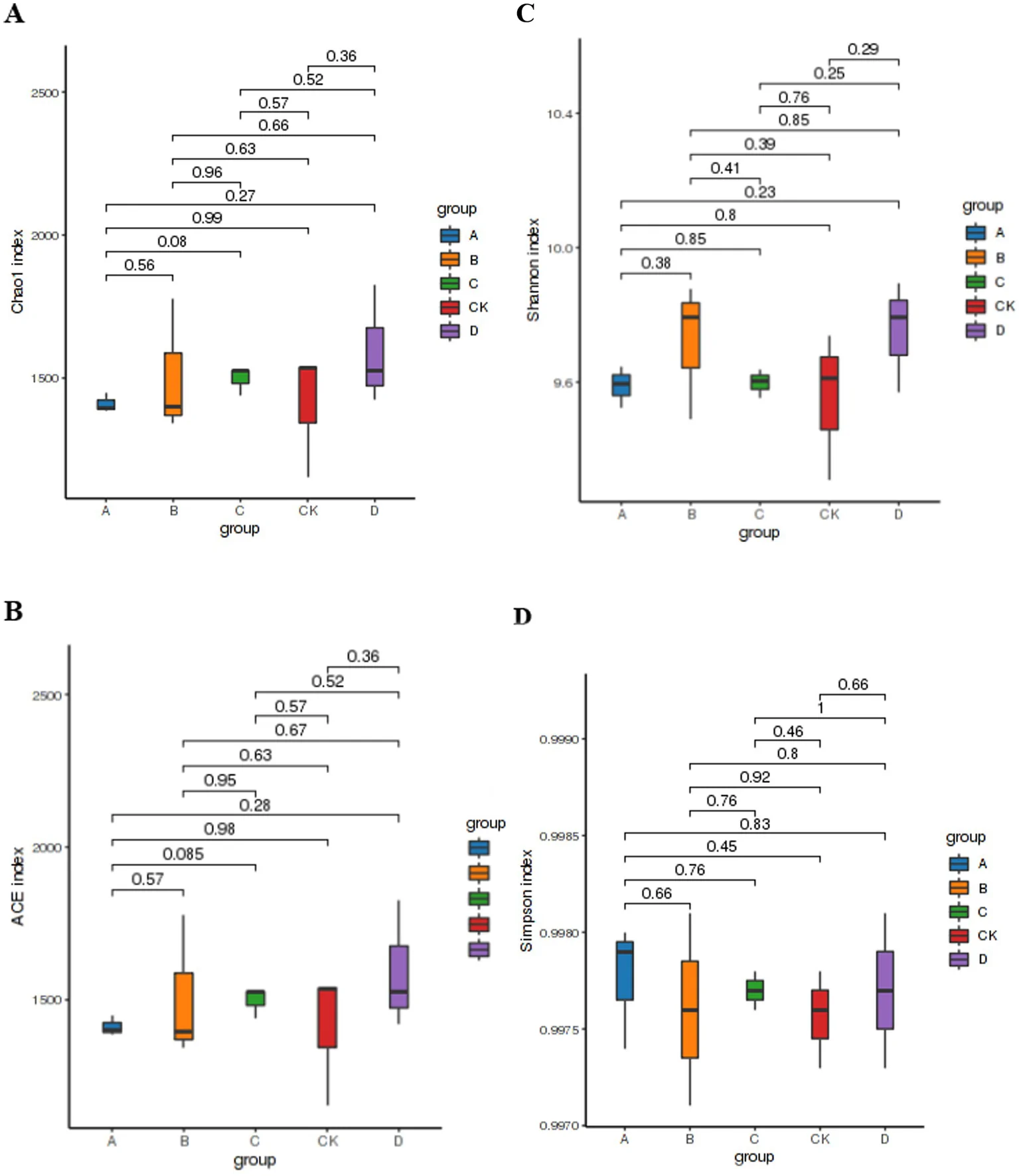
Alpha diversity of rhizosphere bacteria communities of *Erigeron breviscapus* by companion cropping. The numeric values above each boxplot indicate adjusted *P*-values for pairwise comparisons across experimental groups.

The alpha diversity analysis of the endophytic fungal communities in various tissues of *E. breviscapus* showed that there were no significant statistical differences in the Chao1 index and ACE index among different treatment groups (Figures 10A,B). Furthermore, when comparing the Shannon and Simpson indices of the five treatment groups, no significant changing trend was observed. There were no significant differences in bacterial richness and diversity among the treatment groups (Figures 10C,D).

### 3.8 Restoration effects of different crops intercropping on soil microbial richness and diversity

The effects of different treatments on the alpha diversity of fungal and bacterial communities in the rhizosphere soil of *E. breviscapus* were significantly different (Table 7). The fungal community was more sensitive to intercropping treatments, with significant differences in species richness, community diversity, and evenness among the different treatments. Specifically, the Chao1, ACE richness, Shannon diversity, and Simpson evenness indices of the fungal community under treatment A were significantly lower than those of the CK group, indicating that the fungal community structure was substantially unbalanced. Under treatment C, the Chao1 and ACE richness indices of the fungal community were significantly higher than those of the CK group, while the community diversity and evenness remained comparable to those of the CK group, indicating a positive regulatory effect on the fungal community. Treatments B and D had relatively weak effects on the fungal community. In sharp contrast to the fungal community, the Chao1, ACE, Shannon, and Simpson indices of the rhizosphere bacterial community under all treatments were not significantly different from those of the CK group, indicating that the bacterial community had low sensitivity to the treatments and remained highly stable as a whole.

**Table 7:** Effects of companion cropping on soil microbial abundance and diversity.

Treatment	Fungi					Bacteria			
	Chao1 Index	Shannon Index	ACE	Simpson		Chao1 Index	Shannon Index	ACE	Simpson
CK	446 ± 60.81ab	7.99 ± 0.05a	446 ± 60.81ab	0.9927 ± 0.0007a		1,409 ± 182.20a	9.55 ± 0.18a	1,409 ± 181.90a	0.9976 ± 0.0002a
A	386 ± 34.87b	5.77 ± 0.23b	386 ± 34.87b	0.9545 ± 0.0068c		1,411 ± 27.81a	9.59 ± 0.05a	1,413 ± 27.56a	0.9978 ± 0.0003a
B	483 ± 19.57ab	6.28 ± 0.09b	483 ± 19.43ab	0.9658 ± 0.0034bc		1,505 ± 193.41a	9.72 ± 0.17a	1,506 ± 193.18a	0.9976 ± 0.0004a
C	541 ± 91.26a	7.61 ± 0.31a	541 ± 91.26a	0.9858 ± 0.0063ab		1,497 ± 41.51a	9.60 ± 0.03a	1,497 ± 41.51a	0.9977 ± 0.0001a
D	471 ± 56.79ab	7.31 ± 0.94a	471 ± 56.90ab	0.9757 ± 0.0223abc		1,591 ± 171.96a	9.75 ± 0.14a	1,592 ± 171.75a	0.9977 ± 0.0003a

Values are the mean ± standard deviation (SD) of three replicates. Different letters in the same column indicate significant differences (*p* < 0.05).

### 3.9 Correlation between soil microorganisms and soil properties in intercropping of different crops

**Figure 11:**
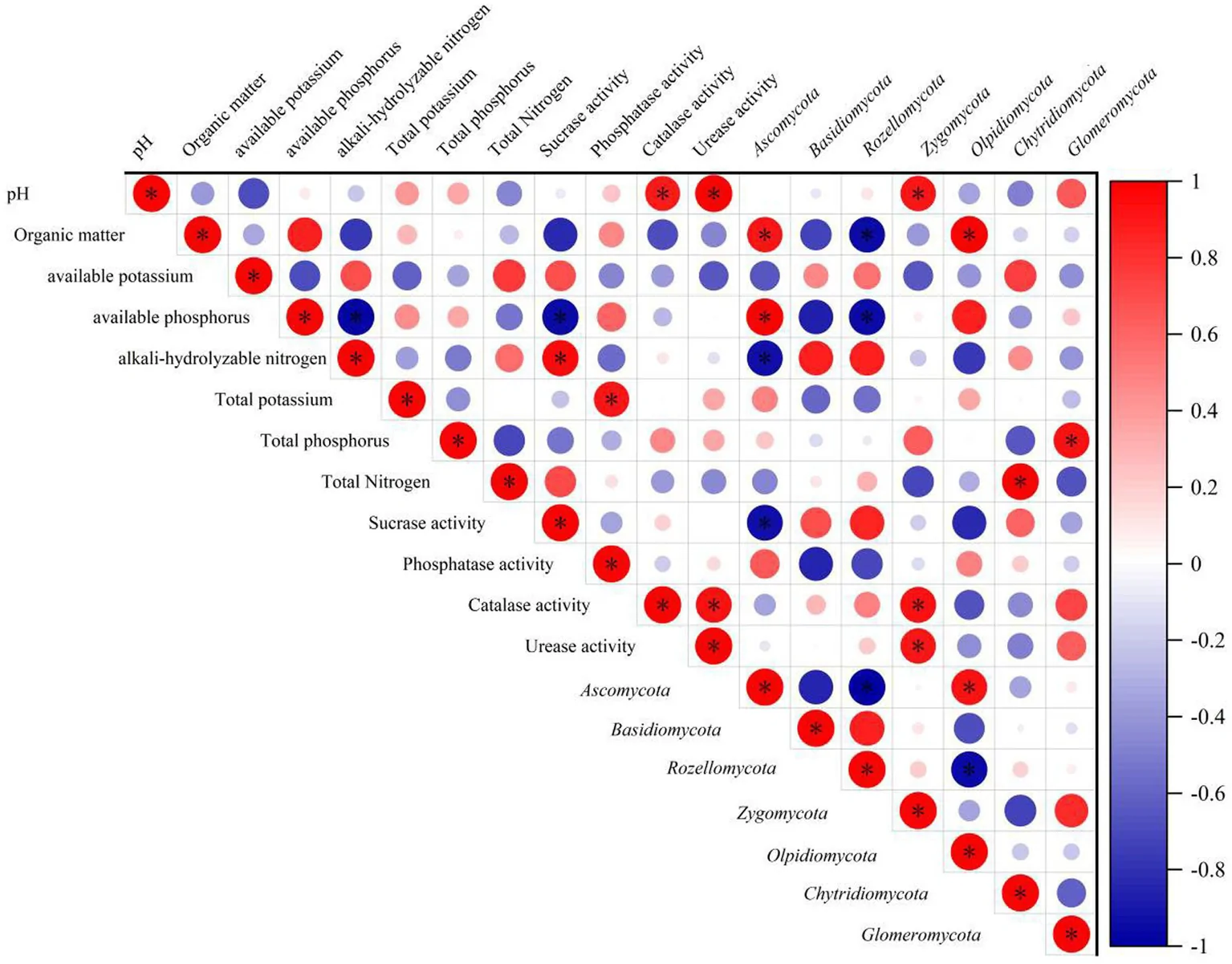
Correlation between soil fungi and properties in different companion cropping. The circle size and color intensity are related to each coefficient. Positive correlations and negative correlations are displayed in purple and blue, respectively. * Represents a correlation at the 0.05 level, and ** represents a correlation at the 0.01 level.

**Figure 12:**
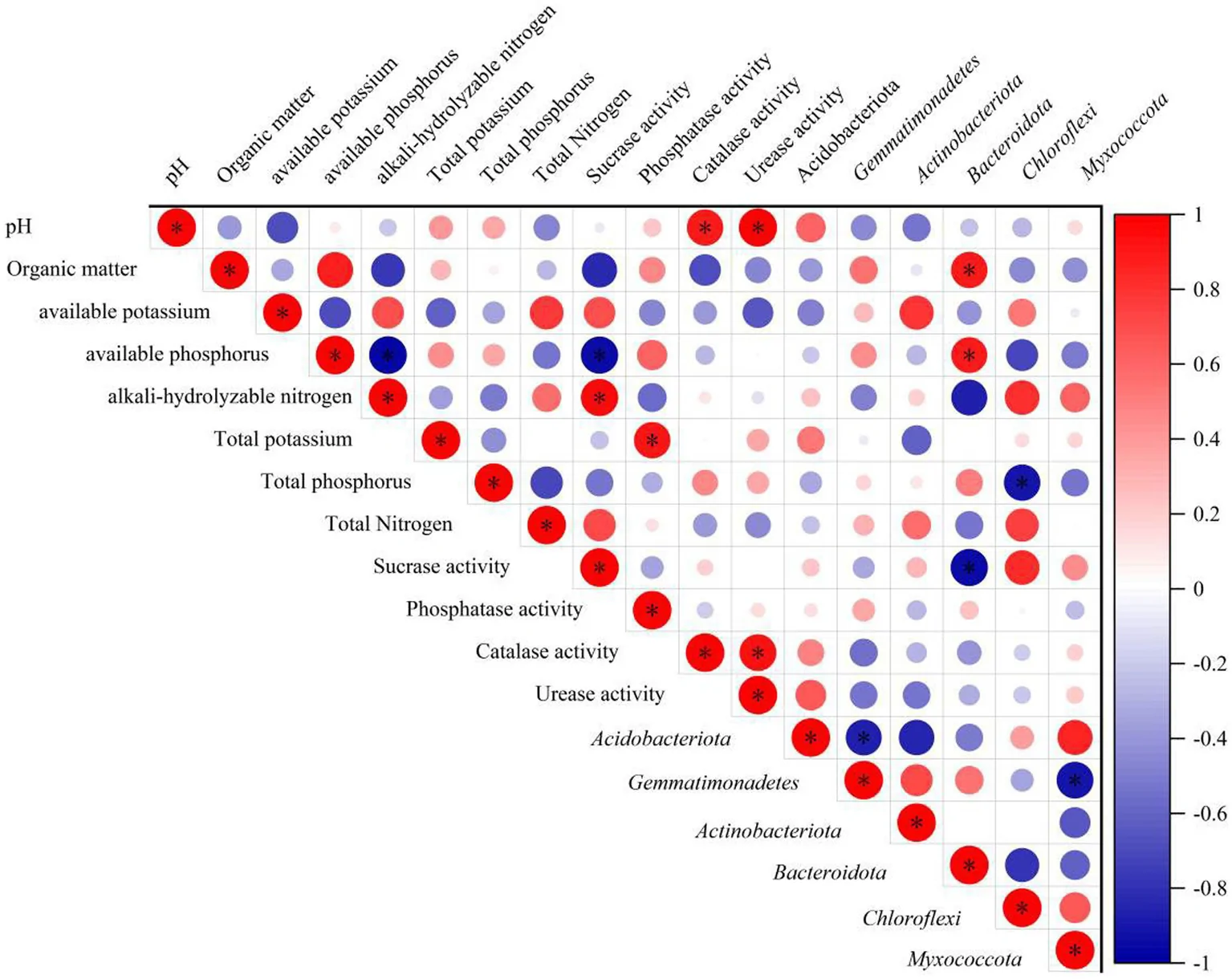
Correlation between soil bacteria and properties in different companion cropping. The circle size and color intensity are related to each coefficient. Positive correlations and negative correlations are displayed in red and blue, respectively. * Represents a correlation at the 0.05 level, and ** represents a correlation at the 0.01 level.

This study examined the relationships between rhizosphere soil fungi and soil properties during the late growth stages of different intercropped crops. At the fungal phylum level (Figure 11), the phylum Olpidiomycota exhibited a significantly negative correlation with sucrase activity and a significantly positive correlation with available phosphorus and organic matter; the phylum Chytridiomycota had a significantly negative correlation with total phosphorus. This pattern was consistent with the phosphorus-limited growth traits of Chytridiomycota fungi reported in prior aquatic microbial surveys (Hanrahan-Tan et al., 2022). Because the growth of Chytridiomycota is limited by phosphorus concentration, rhizosphere environment with low total phosphorus facilitates its proliferation, consistent with the correlations observed in this study. Additionally, the correlation analysis was performed to explore the relationships between rhizosphere soil bacteria, soil properties, and fungal phyla during the late stage of different intercropped crops (Figure 12). The phylum *Proteobacteria* was highly significantly positively correlated with available P and also positively correlated with soil organic matter. On the contrary, this phylum had a significant negative correlation with alkaline hydrolyzable nitrogen and sucrase activity. The phylum *Gemmatimonadota* was significantly negatively correlated with organic matter and available phosphorus. The phylum *Actinobacteriota* was significantly positively correlated with soil pH, catalase activity, and urease activity. The phylum *Bacteroidota* was significantly positively correlated with organic matter; the phylum *Chloroflexi* was significantly positively correlated with available phosphorus; the phylum *Myxococcota* was significantly positively correlated with total phosphorus.

## 4 Discussion

A large number of studies have shown that continuous cropping has a negative impact on the growth and development of plants, significantly reducing crop yield and quality and increasing crop disease incidence, while reasonable intercropping can play a certain role in alleviating continuous cropping obstacles (Tan et al., 2021). The results of this study indicated that different intercropping crops exerted dual effects on the growth and quality of *E. breviscapus*, with distinct crop-specific intercropping responses. In terms of active ingredient accumulation, intercropping with garlic and broad beans significantly improved the total flavonoid content of *E. breviscapus*, and corn intercropping also exhibited a positive promotion effect on flavonoid accumulation. These phenomena may be attributed to the interspecific facilitation effect of intercropping crops. Reasonable intercropping can optimize the field microenvironment, regulate rhizosphere soil nutrient supply and microbial community structure, and further activate the secondary metabolic pathway of *E. breviscapus*, thereby promoting the synthesis and accumulation of flavonoid active substances. Notably, despite the flavonoid improvement effect of garlic intercropping, individual intercropping treatment inhibited the accumulation of key active ingredients of *E. breviscapus*. Specifically, the scutellarin content of *E. breviscapus* under all intercropping regimes was lower than that under monoculture. This variation in active ingredient accumulation suggests that flavonoids and scutellarin have different metabolic response mechanisms to intercropping stress and interspecific interaction. In addition, the reduction of the scutellarin content under intercropping may be closely related to field planting location and microenvironmental heterogeneity, including differences in light transmittance, soil water and heat conditions, and interspecific competition intensity in different planting zones, which interfere with the biosynthesis of scutellarin.

In terms of the aboveground morphological growth of *E. breviscapus*, no significant differences were observed between the four intercropping treatments and the CK group at the initial planting stage. This indicates that the early growth of *E. breviscapus* was less affected by interspecific competition and that the seedlings had strong environmental adaptability in the early growth stage. With the progression of growth, interspecific resource competition gradually intensified. Intercropping with *B. juncea* L., corn, and broad beans effectively increased the plant height of *E. breviscapus*, probably reflecting a shade avoidance response of plants to intercropping shading conditions. To obtain sufficient light resources, *E. breviscapus* promoted longitudinal stem elongation, resulting in increased plant height. However, such compensatory growth affected vegetative leaf growth: the leaf number, leaf length, and leaf width of *E. breviscapus* were significantly reduced under these three intercropping treatments. The inhibited leaf development further limited the photosynthetic area and photosynthetic efficiency of *E. breviscapus*, which was not conducive to the accumulation of photosynthates and vegetative growth.

Overall, monoculture was more favorable to the aboveground morphological growth of *E. breviscapus* than intercropping systems. Most intercropping treatments failed to improve the comprehensive growth status of *E. breviscapus* and even inhibited leaf growth and development. The dual effects of intercropping on the growth and quality of *E. breviscapus* fully reflect the complexity of interspecific interactions in intercropping systems. On the one hand, appropriate intercropping crops can improve the medicinal quality of *E. breviscapus* by promoting flavonoid accumulation; on the other hand, interspecific competition for light, water, and nutrients restricts the vegetative growth of *E. breviscapus* and reduces the content of key active ingredients such as scutellarin. This study implies that screening suitable intercropping crops and optimizing intercropping patterns are essential to balance the growth and quality of *E. breviscapus* and avoid the adverse effects of intercropping while exerting its quality-improving advantages.

Other studies have reported that intercropping *P. grandiflorus* with *Glehnia littoralis* improved both crop quality and yield and reduced root rot incidence (Liu P. F. et al., 2025). The tobacco–soybean intercropping system also achieved the highest soil nutrient replenishment rate, tobacco leaf nutrient density, leaf quality, and profitability (Liu X. L. et al., 2025). However, the results of this study are inconsistent with the findings of previous studies. This difference may be attributed to the height and spacing of the intercropping crops selected in the later growth stage, which may have shaded *E. breviscapus*, reducing its photosynthetic rate and further deteriorating flower quality. For the underground root system of *E. breviscapus*, the root growth was significantly inhibited under all intercropping treatments, and there were significant differences in root diameter and specific root surface area between the intercropping treatments and the control group.

Soil nutrient deficiency and decreased enzyme activity often occur after continuous cropping (Zhang P. P. et al., 2024; Song et al., 2024). Microbial communities and their diversity have significant interactions with soil respiration, organic matter synthesis and decomposition, and nutrient cycling; they are also key components for evaluating soil quality (Iqbal et al., 2025; Yoon et al., 2024). Consistent with the typical continuous cropping obstacles observed in most medicinal plant cultivation, long-term monoculture of *E. breviscapus* significantly altered soil physicochemical properties and enzyme activity in the present study. Compared with the three-year cultivated soil, continuous cropping induced a distinct “decline-then-rise” variation in soil catalase and acid phosphatase activities, as well as soil pH and available phosphorus content. This fluctuating trend indicated that continuous cropping did not cause sustained soil degradation, but triggered a stage-specific soil biochemical response in *E. breviscapus* rhizosphere soil. Notably, soil organic matter and alkali-hydrolyzable nitrogen contents were elevated under continuous cropping conditions. This phenomenon may be explained by the autotoxic and allelopathic effects generated by long-term *E. breviscapus* monoculture. Continuous cropping accumulation of root exudates and residual litter produces autotoxic substances, which not only suppress plant root growth and nutrient absorption capacity but also alter soil biochemical reactions, thereby changing the accumulation characteristics of soil nitrogen and organic matter. Furthermore, the variation in soil enzyme activity and nutrient status observed in this study was closely associated with rhizosphere microbial fluctuations, suggesting that soil microorganisms are crucial mediators regulating soil nutrient cycling and enzyme activity under *E. breviscapus* continuous cropping systems.

Rhizosphere microbial community composition and diversity are core biological factors determining soil ecological stability and nutrient cycling function. The present results demonstrated that both monoculture and intercropping treatments effectively reshaped the rhizosphere microbial richness and diversity of *E. breviscapus*. In comparison with monoculture, most intercropping patterns (excluding *B. juncea* L. intercropping) improved the number of unique bacterial and fungal OTUs in the rhizosphere soil, which effectively enriched rhizosphere microbial unique species resources. These findings verified that reasonable interspecific planting can optimize rhizosphere microbial niche differentiation, increase microbial community specificity, and break the microbial community simplification caused by long-term monoculture of *E. breviscapus*. Nevertheless, the failure of *B. juncea* L. intercropping to increase unique microbial OTUs indicated that the regulatory effect of intercropping on soil microbial communities is highly crop-specific, which may be related to the differences in root exudate components and interspecific competitive relationships of different intercropping crops. Collectively, intercropping can effectively optimize the rhizosphere microbial microenvironment of *E. breviscapus*, which provides a favorable biological foundation for alleviating continuous cropping obstacles and improving soil health.

Intercropping with *B. juncea* L. reduced the richness and diversity of soil fungal communities, while other intercropping treatments (including intercropping with broad beans and corn) increased the Chao1 index and Shannon diversity index of soil bacteria compared with monoculture, among which intercropping with broad beans and corn significantly increased soil bacterial diversity and richness. This finding is consistent with the results of Tang et al. (2024), who found that rape and Chinese milk vetch are suitable intercropping crops for rice.

Dominant bacteria play a crucial role in maintaining the balance of the microbial ecosystem and can change the composition and structure of the microbial community (Taqi et al., 2023; Xiang et al., 2023). In this study, *Ascomycota* and *Basidiomycota* were the dominant fungal phyla in all treatments, with *Ascomycota* being the most abundant. *Ascomycota* are predominantly saprophytic fungi, and some species are important plant pathogens; they can degrade highly resistant organic substances in the soil, thereby improving the potential of crops to absorb nutrients (Luisa et al., 2024). The cultivation of different intercropping crops disturbed the soil, changing the relative abundance of *Proteobacteria*, *Acidobacteriota*, and *Gemmatimonadota*. The relative abundance of *Proteobacteria* and *Acidobacteriota* in the soil under intercropping with *B. juncea* L. and corn was higher than that under continuous cropping, while the relative abundance of *Gemmatimonadota* was lower than that under continuous cropping, indicating that intercropping with *B. juncea* L. and corn shifted the soil bacterial community structure toward nutrient accumulation in *E. breviscapus*.

At the genus level, different intercropping treatments resulted in different microbial community compositions, which may be attributed to root exudates of intercropping crops. Root exudates can alter the soil microbial community of crops. *Fusarium* is a large genus of pathogenic fungi that can infect various plants, causing soft rot, wilting, root rot, and ear rot, thereby reducing plant health and productivity (Qiu et al., 2025). *Sphingomonas* can promote plant growth by producing antibiotics and lytic enzymes that enhance plant resistance to soil-borne pathogens (Asaf et al., 2020). In this study, the relative abundance of *Fusarium* was the lowest under intercropping with broad beans, while the relative abundance of *Sphingomonas* was the highest under intercropping with garlic. These findings indicate that intercropping can effectively reduce the relative abundance of pathogenic fungal genera and increase the relative abundance of beneficial fungal and bacterial genera.

A major limitation of this study is that soil was collected from a 3-year continuous *E. breviscapus* cropping field but tested in greenhouse pots rather than under field conditions. Pot culture greatly changes soil volume, water regime, and environmental stability, leading to rapid shifts in soil physicochemical and biological properties that cannot fully simulate actual field continuous cropping systems. Pot confinement may alter key processes associated with continuous cropping in several ways, root development is restricted by pot volume, leading to abnormal root morphology, increased root density in a limited soil volume, and stronger local accumulation of root exudates and autotoxic compounds compared with field conditions, which may exaggerate allelopathic inhibition or root–soil feedback. Soil microbial communities in pots are sensitive to disturbance, frequent watering, and reduced spatial connectivity; the shifts in bacterial and fungal communities observed in this study may therefore differ in magnitude or composition from those under field continuous cropping. The nutrient dynamics are altered because pots lack field-scale leaching, runoff, and nutrient supply from deeper soil layers, leading to altered mineralization, immobilization, and nutrient availability that do not fully represent nutrient constraints in the field. Accordingly, the results reflect responses to soil with a continuous cropping history under controlled conditions rather than the full complexity of *E. breviscapus* continuous cropping obstacles in the field. Future field-based long-term positioning trials are needed to verify and complement these findings.

## 5 Conclusion

Screening of intercropping crops in this study revealed mixed outcomes for corn and *B. juncea* L. as intercropping partners for continuously cropped *E. breviscapus*. Previous preliminary observations of rhizosphere improvement were offset by measurable detrimental effects on medicinal yield quality and soil fungal biodiversity, indicating that these two species cannot be classified as fully optimal intercropping combinations. Specifically, corn intercropping significantly impaired core medicinal trait performance, scutellarin content was consistently inferior to the monoculture control across all sampling stages, while leaf number and leaf length were both suppressed. Intercropping with *B. juncea* L. generated an unfavorable rhizosphere fungal profile, marked by reduced overall fungal diversity and the minimum number of unique fungal OTUs among all treatments, which may weaken soil disease suppression capacity under long-term cultivation. Critically, this study lacked a weighted multi-criteria decision analysis framework to integrate and prioritize conflicting indicators—plant vegetative growth, bioactive compound accumulation, soil physicochemical fertility, and microbial community richness were assessed independently without unified quantitative weighting. Given these unavoidable trade-offs across agronomic, pharmaceutical, and soil ecological metrics, corn and *B. juncea* L. hold partial potential to mitigate continuous cropping obstacles of *E. breviscapus*, Further long-term field positioning experiments are indispensable to adjust intercropping layout, planting density, and field management measures, reconcile the conflicting advantages and disadvantages found in pots, and develop balanced intercropping technology suitable for large-scale field *E. breviscapus* planting to simultaneously improve soil microecology, plant growth, and medicinal efficacy.

## Data Availability

The original contributions presented in the study are included in the article/Supplementary material, further inquiries can be directed to the corresponding author.
