# Supplementary material for: Effects of different crop intercropping on quality and rhizosphere soil microbial community of Erigeron breviscapus
Source: Front Microbiol. 2026 Aug 17;17:1834232. doi: 10.3389/fmicb.2026.1834232 (PMC13526998; doi:10.3389/fmicb.2026.1834232)
Supplement: Supplementary file 1 [file Table_1.docx]

**TABLE CAPTIONS**

**Table S1** Nutrient content of soil samples of before companion cropping

**Table S2** Enzyme activity of soil samples before companion cropping

**Tab. S1**

| Year | **Treatment** | **pH** | **Organic matter**  **(g/kg)** | **Available potassium**  **(mg/kg)** | **Available phosphorus**  **(mg/kg)** | **Alkali-hydrolyzable nitrogen**  **(mg/kg)** | **Total potassium(g/kg)** | **Total phosphorus(g/kg)** | **Total Nitrogen**  **(g/kg)** |
| --- | --- | --- | --- | --- | --- | --- | --- | --- | --- |
| **2022.12** | **Continuous cropping soil for three years** | 6.47 | 14.54 | 464.38 | 10.25 | 8.40 | 14.07 | 0.30 | 1.35 |
| **2023.01** | **Soil before Continuous cropping** | 6.62 | 13.94 | 471.81 | 4.79 | 5.13 | 12.71 | 0.26 | 1.92 |

**Tab. S2**

| Year | **Treatment** | **Sucrase activity [mg/(g·24h)]** | **Urease activity**  **[mg/(g·24h)]** | **Catalase activity**  **(mL/g)** | **Acid phosphatase activity**  **[mg/(g·24h)]** |
| --- | --- | --- | --- | --- | --- |
| **2022.12** | **Continuous cropping soil for three years** | 17.48 | 0.22 | 3.34 | 0.88 |
| **2023.01** | **Soil before Continuous cropping** | 17.08 | 0.18 | 3.13 | 0.94 |
